# The Impact of Eye Closure on Anticipatory α Activity in a Tactile Discrimination Task

**DOI:** 10.1523/ENEURO.0412-21.2021

**Published:** 2022-01-18

**Authors:** Hesham A. ElShafei, Corinne Orlemann, Saskia Haegens

**Affiliations:** 1 Donders Institute for Brain, Cognition and Behavior, Radboud University, Nijmegen 6525 EN, The Netherlands; 2 Department of Psychiatry, Columbia University, New York, NY 10032; 3 Division of Systems Neuroscience, New York State Psychiatric Institute, New York, NY 10032

**Keywords:** α oscillations, anticipatory attention, eye closure, functional inhibition, MEG, tactile discrimination

## Abstract

One of the very first observations made regarding α oscillations (8–14 Hz), is that they increase in power over posterior areas when awake participants close their eyes. Recent work, especially in the context of (spatial) attention, suggests that α activity reflects a mechanism of functional inhibition. However, it remains unclear how eye closure impacts anticipatory α modulation observed in attention paradigms, and how this affects subsequent behavioral performance. Here, we recorded magnetoencephalography (MEG) in 33 human participants performing a tactile discrimination task with their eyes open versus closed. We replicated the hallmarks of previous somatosensory spatial attention studies: α lateralization across the somatosensory cortices as well as α increase over posterior (visual) regions. Furthermore, we found that eye closure leads to (1) reduced task performance; (2) widespread increase in α power; and (3) reduced anticipatory visual α modulation (4) with no effect on somatosensory α lateralization. Regardless of whether participants had their eyes open or closed, increased visual α power and somatosensory α lateralization improved their performance. Thus, we provide evidence that eye closure does not alter the impact of anticipatory α modulations on behavioral performance. We propose there is an optimal visual α level for somatosensory task performance, which can be achieved through a combination of eye closure and top-down anticipatory attention.

## Significance Statement

α Oscillations are dominant when awake participants have their eyes closed. Furthermore, α is known to modulate with anticipatory attention, and has been ascribed a role of active functional inhibition. Surprisingly, the link between anticipatory α and eye closure remains unclear. Here, we collected magnetoencephalography (MEG) data while human participants performed a tactile discrimination task either with their eyes open or closed. Eye closure led to a widespread increase in α power, and affected anticipatory visual α modulation but not somatosensory α lateralization. Importantly, eye closure did not affect the correlation between α and task performance. Our findings provide novel insights into how eye closure impacts anticipatory α modulation, and how optimal α levels for task performance can be achieved differently.

## Introduction

Since the discovery of the cortical α rhythm by Hans Berger almost a century ago (Berger, 1929), it has been known that a general increase of posterior (visual) α power occurs when awake participants close their eyes (Adrian and Matthews, 1934). While traditionally the α rhythm was associated with a state of cortical idling (Pfurtscheller et al., 1996), more recent work suggests that α activity reflects a mechanism of functional inhibition (Klimesch et al., 2007; Jensen and Mazaheri, 2010; Foxe and Snyder, 2011; Haegens et al., 2011). In support of such an inhibitory mechanism, visual spatial attention is known to modulate α activity in a lateralized fashion: α decreases contralateral to the attended location (Sauseng et al., 2005) and increases contralateral to the ignored location, presumably to suppress distracting input (Worden et al., 2000; Kelly et al., 2009; Wöstmann et al., 2019). This lateralized α activity correlates with visual detection performance (Thut et al., 2006; Händel et al., 2011). Similar patterns have been observed for the auditory (Banerjee et al., 2011; Frey et al., 2014; Strauß et al., 2014; Wöstmann et al., 2016) and somatosensory domains (Jones et al., 2010; Anderson and Ding, 2011; Haegens et al., 2011, 2012).

Importantly, in our previous tactile spatial attention work, we found that somatosensory α lateralization was accompanied by an anticipatory increase of visual α power, which positively correlated with tactile discrimination performance. We interpreted this visual α increase to reflect a general inhibition of visual processing to improve tactile performance (Haegens et al., 2010, 2012). An obvious follow-up question is whether a similar visual α increase, and accompanying tactile performance improvement, could be achieved by closing the eyes. Or, in other words, does the anticipatory task-related visual α modulation stem from the same underlying sources as eye-closure-related α modulation? Another question is how eye-closure-induced α increase relates to α lateralization patterns observed in the context of spatial attention.

Anecdotally, eye closure enhances the concentration on other sensory modalities by suppressing processing of visual input (Glenberg et al., 1998). Eye closure has been shown to boost stimulus responses in somatosensory areas (Brodoehl et al., 2015; Götz et al., 2017), with mixed findings regarding impact on behavioral performance. To date, the relationship between eye-closure effects and anticipatory α modulation has only been investigated in the context of auditory attention, Wöstmann et al. (2020) showed that eye closure increases the general power of α oscillations, as well as the modulation of α during an auditory attentional task; however, this had no impact on behavioral performance.

Here, we asked whether and how eye-closure-induced α modulations interact with anticipatory α modulations and associated behavioral performance effects. We recorded magnetoencephalography (MEG) while participants performed an adapted version of the tactile discrimination task from Haegens et al. (2011), during eyes-open (EO) and eyes-closed (EC) conditions. First, we asked whether the often-reported eye-closure-related power increase extends beyond visual α. Next, we compared the previously reported anticipatory α modulations, i.e., somatosensory α lateralization and visual α increase (Haegens et al., 2012), between eye conditions and asked how they interact with the eye-closure-related power increase. Finally, we asked whether the relationship between these α modulations and task performance differs across eye conditions; specifically, whether visual α increase (which we previously interpreted as inhibition of visual processing) is behaviorally relevant in the absence of visual input.

## Materials and Methods

### Participants

Participants were 34 healthy adults (age: M = 25, SD = 3.86, range = 20–33 years; 18 female; 30 right-handed, two left-handed, two ambidextrous) without neurologic or psychiatric disorders, who reported normal hearing and normal or corrected-to-normal vision. The study was approved by the local ethics committee (CMO 2014/288 “Imaging Human Cognition”) and in accordance with the Declaration of Helsinki. Participants gave written informed consent and were remunerated for their participation. One participant was excluded from analysis because of poor data quality.

### Experimental design

**Figure 1.**
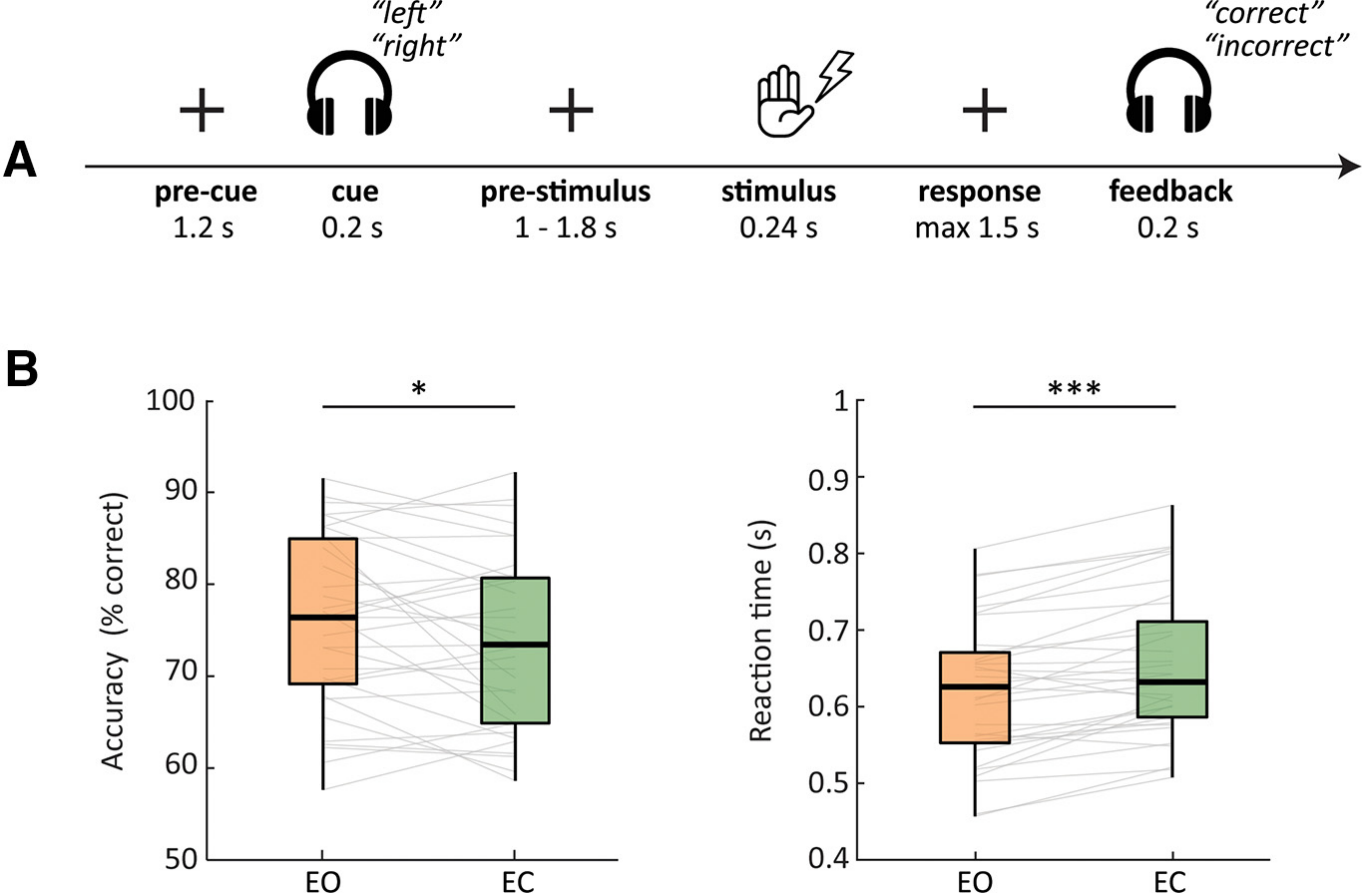
Experimental paradigm and behavioral results. ***A***, Participants performed a tactile stimulus discrimination task where a 100% valid auditory cue directed attention either to their right or left hand in an EO and an EC condition. Participants had to discriminate between two target frequencies, presented as electrical pulse trains to the cued thumb. ***B***, Accuracy (left panel) and reaction time (right) for the EO and EC conditions. Behavioral performance was significantly worse when participants had their eyes closed both in terms of lower accuracy and slower RT; *p < 0.05, **p < 0.01, ***p < 0.001. Within each boxplot, the horizontal line represents the median, the box delineates the area between the first and third quartiles (interquartile range).

Participants performed a tactile discrimination task (Fig. 1; task adapted from Haegens et al., 2011), while their brain activity was recorded using MEG. Participants received an electrical stimulus (pulse train of a low or high frequency) to either the right or left thumb. Participants were instructed to determine as fast and accurately as possible whether the perceived stimulus was of low or high frequency, responding via button press with their right index finger (left button press indicated the low frequency; right button press indicated the high frequency). Before the stimulus presentation, an auditory cue (verbal “right” or “left”) directed participants’ attention to either their right or left hand. Spatial cues were always valid. Each trial started with a precue interval of 1.2 s followed by the auditory cue (0.2 s), a jittered 1–1.8 s prestimulus interval, the tactile stimulus (0.24-s pulse train), a response window of maximum 1.5 s, and finally auditory feedback indicating whether the answer was correct or incorrect.

Participants performed this task under two conditions: an EO and an EC condition. Conditions were presented in a counter-balanced block-design of four blocks per condition with 76 trials each, resulting in a total of 304 trials per condition. During the EO condition, participants were instructed to fixate on a fixation cross in the middle of the screen. For the EC condition, participants kept their eyes closed for the duration of the block. After each block, participants were presented with a short questionnaire to rate their sleepiness level (very sleepy, sleepy, awake, very awake). This was done to check for potential confound of decreased arousal with eye closure. Before the experiment, participants performed four training blocks (two per condition, 12 trials per block), during which they were familiarized with the task.

### Stimulus presentation

We used the same setup as in Haegens et al. (2011): electrical stimuli were delivered with two constant-current high-voltage stimulators (Digitimer Ltd, Model DS7A) to the right and left thumb. The intensity (M_right_ = 6.4 mA, range = 3.9–9.5 mA; M_left_ = 5.5 mA, range = 3.2–9.9 mA) of the 0.2-ms electric pulses was set to 150% of the participant’s sensory threshold level. This level was established during a practice session before the recordings, for each thumb independently. Low (either 25 or 33.3 Hz) and high frequencies (41.7, 50, or 66.7 Hz) were determined for each participant individually to ensure successful execution of the task, above chance level but below ceiling performance. Auditory cues and feedback (0.2-s length each) were computer-generated and presented binaurally through air-conducting tubes.

### Data acquisition

Whole-head MEG data were acquired at a sampling frequency of 1200 Hz with a 275-channel MEG system with axial gradiometers (CTF MEG Systems, VSM MedTech Ltd.) in a dimly lit magnetically shielded room. Six permanently faulty channels were disabled during the recordings, leaving 269 recorded MEG channels. Three fiducial coils were placed at the participant’s nasion and both ear canals, to provide online monitoring of participant’s head position (Stolk et al., 2013) and offline anatomic landmarks for co-registration. Eye position was recorded using an eye tracker (EyeLink, SR Research Ltd.). Upon completion of the MEG session, participant’s head shape and the location of the three fiducial coils were digitized using a Polhemus 3D tracking device (Polhemus). Anatomical T1-weighted MRIs were obtained during a separate session. To improve co-registration of the MRIs and MEG data, earplugs with a drop of vitamin E were placed at participant’s ear canals during MRI acquisition. These anatomic scans were used for source reconstruction of the MEG signal.

### Preprocessing

MEG data were preprocessed offline and analyzed using the FieldTrip toolbox (Oostenveld et al., 2011) and custom-built MATLAB scripts. The MEG signal was epoched based on the onset of the somatosensory stimulus (*t* = −4–3 s). The data were downsampled to a sampling frequency of 300 Hz, after applying a notch filter to remove line noise and harmonics (50, 100, and 150 Hz). Bad channels and trials were rejected via visual inspection before independent component analysis (Jung et al., 2001) was applied. Subsequently, components representing eye-related and heart-related artefacts were projected out of the data (on average, eight components were removed per participant). Finally, for the resulting data, outlier trials of extreme variance (higher than 2 SDs) were removed. This resulted in an average of 537 (±7 SEM) trials and 268 channels per participant for the reported analyses.

### Spectral analysis

First, we calculated the planar representation of the MEG field distribution from the single-trial data using the nearest-neighbor method. This transformation makes interpretation of the sensor-level data easier as the signal amplitude is typically maximal above a source. Next, we computed spectral representations for two 1-s time windows: the prestimulus window and the precue window (i.e., baseline), aligned to stimulus and cue onset, respectively. Each window was multiplied with a Hanning taper, and power spectra (1–30 Hz; 1-Hz resolution) were computed using a fast Fourier transform (FFT) approach. Additionally, for a time-resolved-representation of the spectral power distribution, we computed time-frequency representations (TFRs) of the power spectra for the full trials per experimental condition. To this end, we used an adaptive sliding time window of five cycles length per frequency (Δ*t* = 5/f; 20-ms step size).

### α Peak frequency

In order to investigate how eye closure impacts α activity we computed the individual α peak frequencies for each participant, separately for occipital and centroparietal sensor-level regions of interest (ROIs), and separately for the EO and EC conditions. We determined participants’ peak frequencies within a broad α range (7–14 Hz) during the prestimulus interval (−1–0 s). As intraindividual α peaks did not significantly vary with condition (*F*_(1,32)_ = 0.46, *p* = 0.5, ANOVA) or ROI (*F*_(1,32)_ = 1.04, *p* = 0.31), nor their interaction (*F*_(1,32)_ = 0.17, *p* = 0.67), we computed one average peak for each participant (M* *=* *10 Hz, range = 7–13 Hz). Using individual α peak frequency allows taking into account interindividual variability and provides a more accurate estimation of α activity than when using a fixed frequency band (Haegens et al., 2014). All further analysis was computed using these individual α peaks, with spectral bandwidth of ±1 Hz, unless indicated otherwise.

### Statistical analysis

In order to investigate whether power differences between the EO and the EC conditions were significant, we used nonparametric cluster-based permutation tests (Maris and Oostenveld, 2007). In brief, this test first calculates paired *t* tests for each sensor at each time and/or frequency point, which are then thresholded at *p* < 0.05 and clustered on the basis of spatial, temporal, and/or spectral adjacency. The sum of *t* values within each cluster is retained, and the procedure is repeated 1000 times on permuted data in which the condition assignment within each individual is randomized. On each permutation, the maximum sum is retained. Across all permutations, this yields a distribution of 1000 maximum cluster values. From this distribution, the probability of each empirically observed cluster statistic can be derived (evaluated at α = 0.05).

We used this permutation test to investigate the impact of eye closure on (1) overall oscillatory power, by contrasting power in the prestimulus interval between eye conditions; (2) anticipatory visual α activity, by contrasting prestimulus baseline-normalized power between eye conditions, for each cue separately; and (3) somatosensory α activity, by contrasting the prestimulus attention modulation index, calculated as (attention-left – attention-right)/(attention-left + attention-right) between eye conditions.

In order to investigate the impact of prestimulus α activity on behavioral performance, we focused our analysis on visual and somatosensory ROIs that were defined in sensor space. For the somatosensory ROIs, our selection was data-based, i.e., per hemisphere we selected 10 sensors with the maximum evoked response to contralateral tactile stimulation. For the visual ROIs, as our design lacked visual stimuli, our selection included 10 left and 10 right occipital sensors. One participant was excluded from analysis because of poor data quality. Note that for α power in the visual ROIs, we use the term “absolute” modulation to denote overall non-baseline-normalized power in the prestimulus window, while the term “anticipatory” denotes the baseline-normalized power in the same prestimulus window.

### α Lateralization index

To capture the relative prestimulus somatosensory α distribution over both hemispheres in one measure, we computed a lateralization index of α power (Thut et al., 2006; Haegens et al., 2011) for each participant, using individual somatosensory ROIs: α lateralization index = (α-ipsilateral – α-contralateral)/(α-ipsilateral + α-contralateral). This index gives positive values if α power is higher over the ipsilateral hemisphere and/or lower over the contralateral hemisphere (with contralateral and ipsilateral sides defined with respect to the spatial cue). Negative values arise if α power activity is lower over the ipsilateral hemisphere and/or higher over the contralateral hemisphere.

### Source reconstruction

In order to localize the generators of the sensor-level spectrotemporal effects, we applied the frequency-domain adaptive spatial filtering technique of dynamical imaging of coherent sources (Gross et al., 2001). For each participant, an anatomically realistic single-shell headmodel based on individual T-1 weighted anatomic images was generated (Nolte, 2003). The brain volume of each individual subject was divided into a grid with a 0.5-cm resolution and normalized toward a template MNI brain using nonlinear transformation. For each grid point, leadfields were computed with a reduced rank, which removes the sensitivity to the direction perpendicular to the surface of the volume conduction model. This procedure ensures that each grid-point represents the same anatomic location across all participants by taking into account the between-subject difference in brain anatomy and head shape.

Data from all conditions of interest were concatenated to compute the cross-spectral density (CSD) matrices (multitaper method; Mitra and Pesaran, 1999). Leadfields for all grid points along with the CSD matrices were used to compute a common spatial filter (i.e., common for all trials and conditions) that was used to estimate the spatial distribution of power for time-frequency windows of interest highlighted in the previous analysis. The source orientation was fixed to the dipole direction with the highest strength.

## Results

### Eye closure impairs performance

Performance over all 33 participants for both eye conditions combined was an average accuracy of 74.4% (SD = 9.96%) and an average reaction time (correct trials only) of 0.64 s (SD = 0.1 s). Participants were more accurate (*t*_(32)_ = 2.32, *p* = 0.023, paired-test, mean EO = 75.7 + 9.9% SD, mean EC = 73.7 ± 9.9% SD) and faster (*t*_(32)_ = −6.8, *p* < 0.001, mean EO = 0.62 ± 0.1 s SD, mean EC = 0.65 ± 0.1 s SD) at discriminating the frequency of the tactile stimuli in the EO condition in comparison to the EC condition (Fig. 1*B*).

Further, we investigated the impact of eye closure (two levels: EC and EO) and block order (four levels: first, second, third, and fourth) on the sleepiness score reported at the end of each block. We found a main effect of eye condition (*F*_(1,26)_ = 9.7, *p* = 0.004, ANOVA), with participants reporting being more awake when they had their eyes open. In addition, we found a main effect of block order (*F*_(3,78)_ = 5.32, *p* = 0.009), with participants reporting being more awake in the first block in comparison to the second (*t*_(26)_ = −3.15, *p* = 0.014, *post hoc* paired *t* test), third (*t*_(26)_ = −3.45 *p* = 0.005) and fourth (*t*_(26)_ = −3.15, *p* = 0.014), with no significant interaction (*F*_(3,78)_ = 1.11, *p* = 0.35). Note that differences in sleepiness scores did not correlate with differences in behavioral performance between eye conditions (RT: *r*_(26)_ = −0.19, *p* = 0.32; accuracy: *r*_(26)_ = 0.22, *p* = 0.25).

### Eye closure boosts widespread oscillatory activity

**Figure 2.**
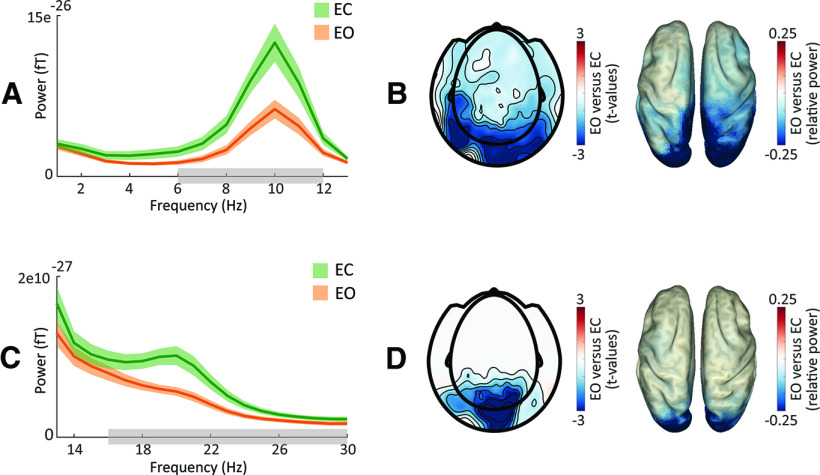
Impact of eye closure on overall power. ***A***, Average absolute occipital power (1–13 Hz) during the prestimulus window (*t* = −1–0 s) for the EC (green) and EO (orange) conditions (shading reflects between-participant SEM). α Power was significantly higher in the EC condition compared with the EO condition. Gray bars indicate significant differences between conditions. ***B***, Topography of significant (masked at *p* < 0.05) cluster *t* values for the α band for EO versus EC (as marked in ***A***) on sensor level (left panel) and power distribution of these differences in source space (right). ***C***, Same as panel ***A*** for 13–30 Hz. β Power was significantly higher in the EC condition compared with the EO condition. ***D***, Same as panel ***B*** for the β band (as marked in ***C***).

In order to investigate the impact of eye closure on overall oscillatory power, we contrasted power spectra (1–30 Hz) during the prestimulus window between the EO and the EC conditions (Fig. 2). We found that power was higher for EC than EO (cluster-corrected *p* < 0.001), both in the α (6–12 Hz) and in the β range (17–30 Hz). The α cluster was widespread with a spectral peak at 10 Hz, while the β cluster was concentrated toward posterior sensors, showing the highest difference between conditions around 20 Hz. While in this study we focused on α activity, as a control we compared event-related fields (ERFs) between eye conditions and found no differences (cluster-corrected *p* > 0.5; test included all sensors, *t* = 0–0.6 s).

### Eye closure impacts anticipatory visual α modulation

**Figure 3.**
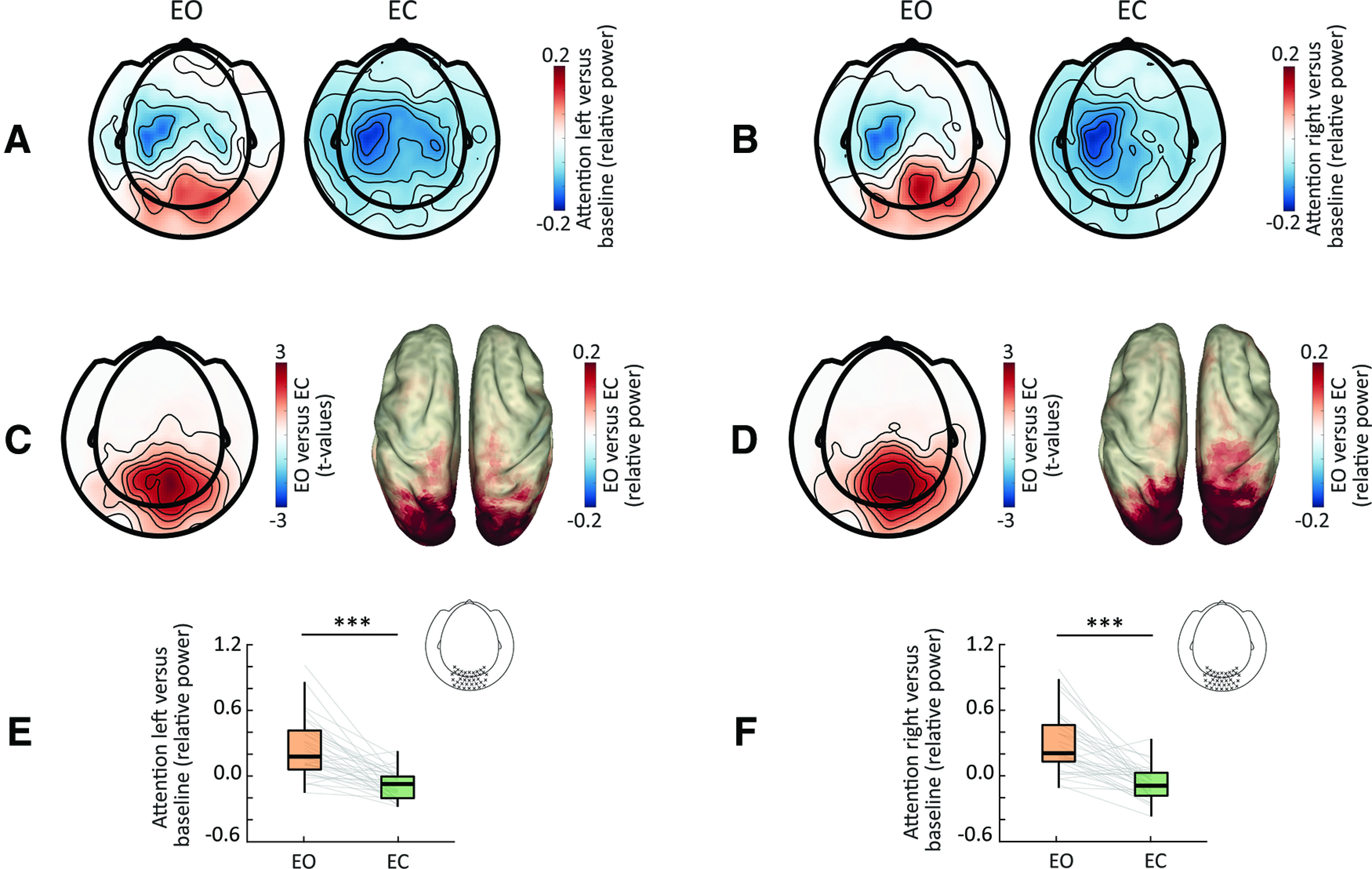
Impact of eye closure on anticipatory visual α modulation. ***A***, Topography of the normalized prestimulus α power modulation for the attention-left condition (i.e., prestimulus window vs baseline) for EO (left panel) and EC (right). ***B***, Same as ***A*** for the attention-right condition. ***C***, Topography of significant (masked at *p* < 0.05) cluster *t* values for EO versus EC for the attention-left condition on sensor level (left panel), and power distribution of these differences in source space (right). ***D***, Same as ***C*** for the attention-right condition. ***E***, Normalized occipital prestimulus α power for the attention-left condition (included sensors marked in topography inset), showing significant difference between eye conditions. ***F***, Same as ***E*** for the attention-right condition; *p < 0.05, **p < 0.01, ***p < 0.001.

In order to investigate the impact of eye closure on anticipatory α modulation (averaged across attention-left and attention-right conditions), we first contrasted α power between the prestimulus and the baseline (i.e., precue) windows. We found a prestimulus decrease of α power over left central sensors versus baseline, for both EO and EC conditions (cluster-corrected *p* = 0.005; Fig. 3*A*,*B*). Furthermore, we observed a prestimulus increase of posterior α power (*p* = 0.001), which was exclusive to the EO condition. Next, we directly contrasted the baseline-normalized prestimulus α between EO and EC conditions, separately for each attention condition (i.e., attend left and right). For both attention conditions, we found higher posterior α power in the EO condition compared with the EC condition (cluster-corrected *p* < 0.001; Fig. 3*C*,*D*). This result reflects an increase of visual α power during the prestimulus interval versus baseline in the EO condition, an effect that was absent in the EC condition. Hence, despite an overall increase of α power with eye closure, the anticipatory posterior α modulation during the prestimulus interval was higher for open eyes.

### Eye closure-related and anticipatory α modulations are spatially distinct

**Figure 4.**
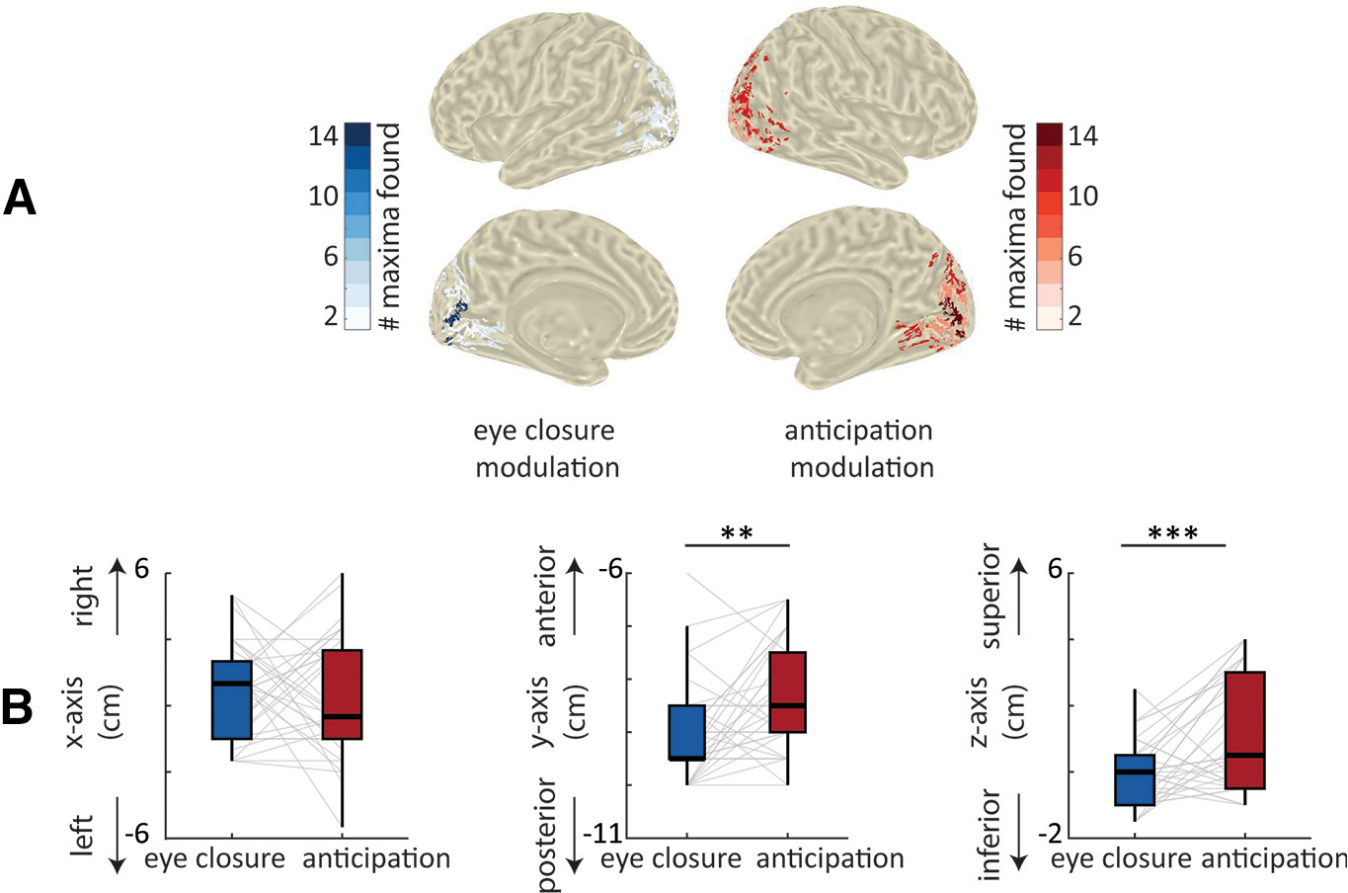
Localization differences between eye-closure and anticipatory α modulations. ***A***, Distribution of the eye-closure (in blue, left) and anticipatory (in red, right) α modulations in posterior (visual) regions in source space. For visualization purposes, maximas from each modulation were transposed on one hemisphere. ***B***, Maxima coordinates along the *x*-axis (left), *y*-axis (middle), and *z*-axis (right); *p < 0.05, **p < 0.01, ***p < 0.001.

To address the question of whether eye-closure-induced modulations and anticipatory α modulations share the same underlying cortical generators (i.e., localize to the same cortical regions), we compared the maxima of these effects in source space. For each participant, we identified the voxel displaying the maximal difference in absolute α power in the EO and the EC conditions, and the voxel displaying the maximal anticipatory prestimulus α power modulation. We then contrasted the *x*-, *y*-, and *z*-coordinates of these maxima using paired *t* tests. We found that maxima differed in their distribution along the *y*-axis (*t*_(32)_ = −2.83, *p* = 0.007 paired *t* test) and the *z-*axis (*t*_(32)_ = −3.7, *p* < 0.001). In other words, maxima of the anticipatory α modulations were located more anterior and superior in comparison to the eye-closure-induced modulations (Fig. 4), with no differences in the distribution along the *x*-axis (i.e., left vs right; *t*_(32)_ = 0.36, *p* = 0.71). While this points to distinct cortical generators for eye-closure and anticipatory α modulations, we are cognizant of the inherent limitation of MEG source localization as well as interindividual variability and anatomic differences across participants; invasive techniques might be required to conclusively resolve this matter.

### Eye closure does not impact somatosensory α modulation

**Figure 5.**
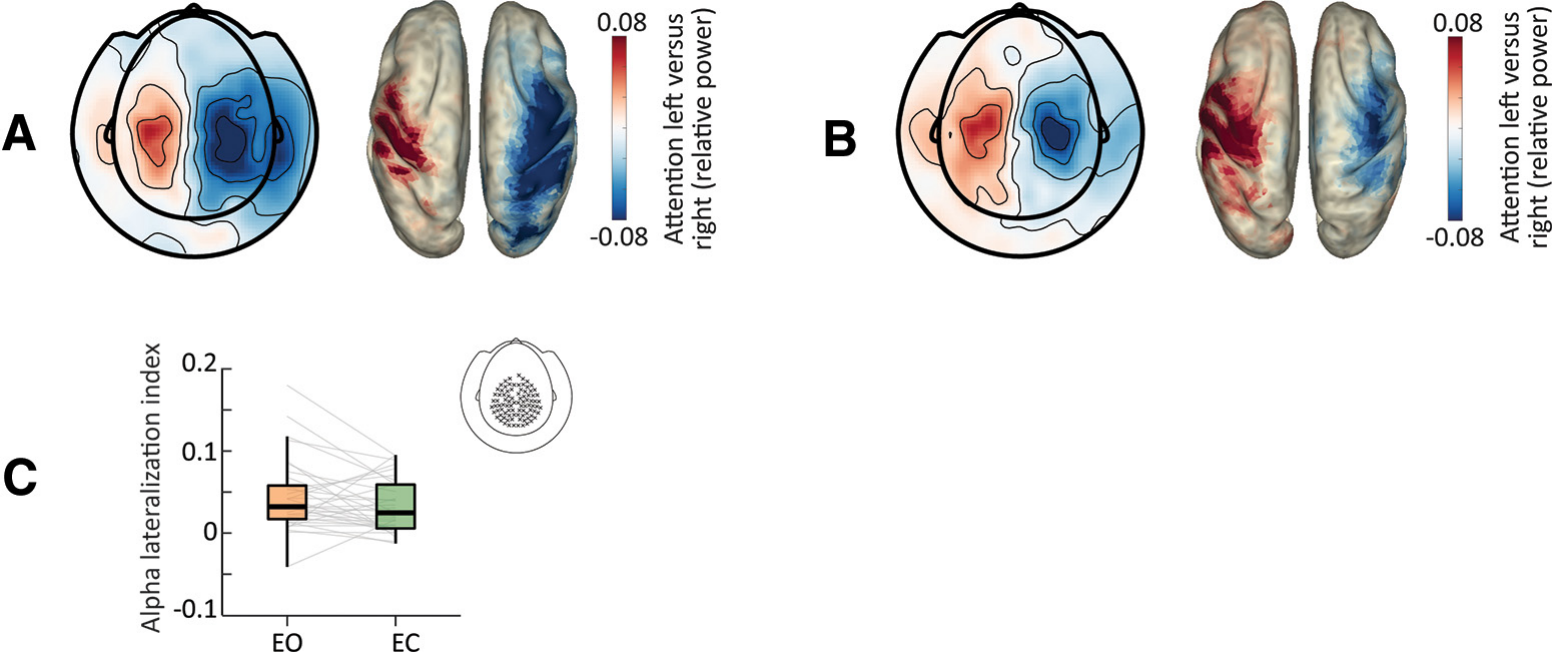
Impact of eye closure on somatosensory α lateralization. ***A***, Topography of the attention-left versus attention-right anticipatory α power modulation for the EO condition (left panel), and power distribution of this modulation in source space (right). This modulation localizes to somatomotor regions with higher α power in ipsilateral and lower α power in contralateral regions. ***B***, Same as ***A*** for the EC condition. ***C***, Prestimulus α lateralization index (included sensors marked in topography inset), showing no significant difference between eye conditions.

In order to investigate how eye closure impacts anticipatory somatosensory α modulation, we contrasted the prestimulus attention modulation index [calculated as (attention-left – attention-right)/(attention-left + attention-right)] between EO and EC conditions. While there was a significant attention modulation, i.e., a pattern of lateralized sensorimotor α power (left increase *p* = 0.007; right decrease *p* < 0.001) when contrasting left versus right attention conditions, no significant differences were found between eye conditions (*p* = 0.34; Fig. 5). Thus, while both overall and anticipatory visual α activity differed between eye conditions, anticipatory somatosensory α modulation was not affected by eye closure.

### Eye closure does not impact the link between anticipatory α and behavioral performance

Finally, we investigated the impact of eye closure on the link between prestimulus α modulation and behavioral performance. First, we analyzed the relationship between prestimulus visual α power, both absolute (non-baseline normalized) and anticipatory (baseline-normalized) modulations, and performance, by binning the data based on correct versus incorrect responses, and fast versus slow RTs (divided by a median split).

**Figure 6.**
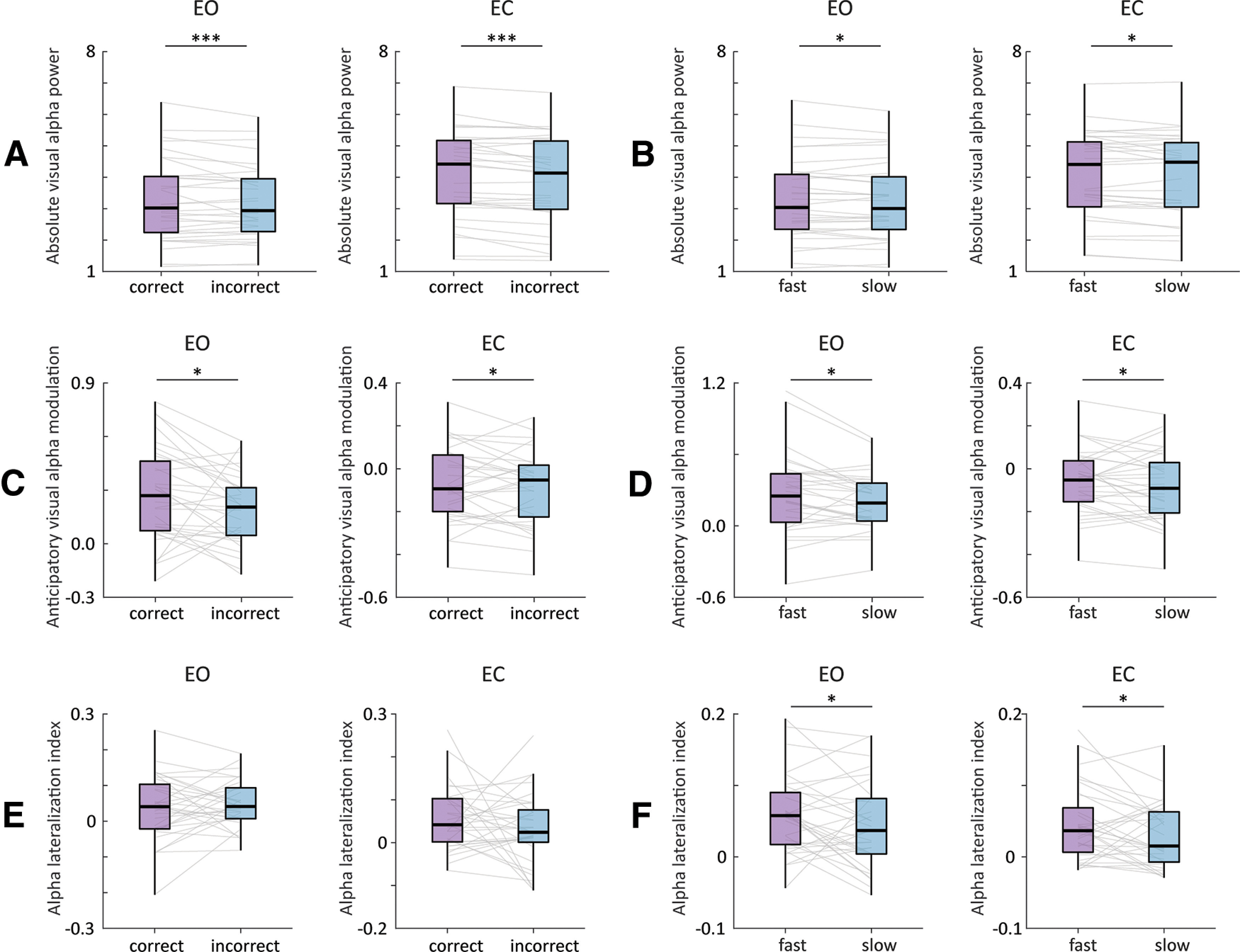
Impact of eye closure on the relationship between α and performance. ***A***, Absolute (non-baseline corrected) prestimulus visual α power in EO (left panel) and EC (right panel) conditions for correct versus incorrect trials. Absolute visual α power was higher for correct trials, regardless of eye condition. ***B***, Same as ***A*** for fast versus slow trials. Absolute visual α power was higher for fast trials, regardless of eye condition. ***C***, Same as ***A*** for anticipatory visual α modulation (baseline corrected) in EO (left panel) and EC (right panel) conditions for correct versus incorrect trials. Anticipatory visual α power was higher for correct trials, regardless of eye condition. ***D***, Same as ***C*** for fast versus slow trials. Anticipatory visual α power was higher for fast trials, regardless of eye condition. ***E***, Same as ***C*** for somatosensory α lateralization index. No significant differences were found between conditions. ***F***, Same as ***E*** for fast versus slow trials. Somatosensory α lateralization was higher for fast trials, regardless of eye condition; *p < 0.05, **p < 0.01, ***p < 0.001.

For absolute visual α power and accuracy (Fig. 6*A*), we found a significant main effect of accuracy (*F*_(1,31)_ = 15.2, *p* < 0.001, ANOVA) with absolute visual α power being higher in correct trials in comparison to incorrect trials. In addition, we found a significant main effect of eye condition (*F*_(1,31)_ = 26.92, *p* < 0.001) and no significant interaction between eye condition and accuracy (*F*_(1,31)_ = 1.15, *p* = 0.29). For absolute visual α power and RT (Fig. 6*B*), we found a significant main effect of RT (*F*_(1,31)_ = 6.11, *p* = 0.02, ANOVA) with absolute visual α power being higher in fast trials in comparison to slow trials. In addition, we found a significant main effect of eye condition (*F*_(1,31)_ = 31.53, *p* < 0.001) and no significant interaction between eye condition and RT (*F*_(1,31)_ = 0.65, *p* = 0.42). In sum, absolute visual α power predicted more accurate and faster responses, regardless of eye condition.

For anticipatory visual α power and accuracy (Fig. 6*C*), we found a significant main effect of accuracy (*F*_(1,31)_ = 4.84, *p* = 0.035, ANOVA) with anticipatory visual α power being higher in correct trials in comparison to incorrect trials. In addition, we found a significant main effect of eye condition (*F*_(1,31)_ = 69.88, *p* < 0.001) and no significant interaction between eye condition and accuracy (*F*_(1,31)_ = 1.77, *p* = 0.19). For anticipatory visual α power and RT (Fig. 6*D*), we found a significant main effect of RT (*F*_(1,31)_ = 7.39, *p* = 0.01, ANOVA) with anticipatory visual α power being higher in fast trials in comparison to slow trials. In addition, we found a significant main effect of eye condition (*F*_(1,31)_ = 41.21, *p* < 0.001) and no significant interaction between eye condition and RT (*F*_(1,31)_ = 1.04, *p* = 0.31). In sum, anticipatory visual α modulation predicted more accurate and faster responses, regardless of eye condition.

For somatosensory α lateralization and accuracy (Fig. 6*E*), we did not find a significant main effect of accuracy (*F*_(1,31)_ = 0.39, *p* = 0.53, ANOVA) nor a significant main effect of eye condition (*F*_(1,31)_ = 0.001, *p* = 0.98), nor a significant interaction between eye condition and accuracy (*F*_(1,31)_ = 1.19, *p* = 0.28). For somatosensory α lateralization and RT (Fig. 6*F*), we found a significant main effect of RT (*F*_(1,31)_ = 5.31, *p* = 0.027, ANOVA) with somatosensory α lateralization being higher for faster trials. We found neither a significant main effect of eye condition (*F*_(1,31)_ = 2.47, *p* = 0.12) nor a significant interaction between eye condition and RT (*F*_(1,31)_ = 0.001, *p* = 0.98). In sum, somatosensory α lateralization predicted faster responses, regardless of eye condition.

## Discussion

In a follow-up on our previous work (Haegens et al., 2010, 2011, 2012), we investigated how eye-closure-related α modulations interact with anticipatory α dynamics and subsequent behavioral performance during a tactile spatial attention task. We found that task performance was reduced with eye closure. While eye closure led to a widespread increase in α power, this only affected anticipatory visual α modulation, with somatosensory α lateralization being the same across EO and EC conditions. Regardless of whether participants had their eyes open or closed, increases in visual α power and somatosensory α lateralization improved their performance.

### Eye closure impacts overall state

Participants were less accurate and slower to discriminate tactile stimuli when their eyes were closed. While there have been several reports of a positive impact of eye closure on performance (e.g., perceptual sensitivity, Brodoehl et al., 2015; memory retrieval, Vredeveldt et al., 2011; Parker and Dagnall, 2020), other studies have reported no effects (e.g., memory retrieval, Bastarrika-Iriarte and Caballero-Gaudes, 2019; selective attention, Wöstmann et al., 2020) or negative impact (somatosensory discrimination, Götz et al., 2017). Differences in paradigms (attention vs memory) and sensory modalities (auditory vs somatosensory) between these various reports renders it difficult to define common factors that govern the interaction between eye closure and behavioral performance. Nevertheless, Götz et al. (2017) argue that for tactile perception, eye closure might boost sensitivity but hinder discriminability, possibly because of the dependence of tactile discriminability on extrastriate visual processing (Sathian and Zangaladze, 2002). Following this logic, in our tactile discrimination task eye closure diminishes extrastriate visual processing, leading to worse behavioral performance.

Simultaneous with this behavioral deterioration, and as has been long known (Adrian and Matthews, 1934; Geller et al., 2014; Wöstmann et al., 2020), α power increased with eye closure. This increase was widespread, extending beyond occipital regions, and additionally included frequency ranges neighboring the α band (i.e., θ and β). This observation supports the idea that eye closure does not only reflect a disengagement of visual areas, but rather a cortical state transition (Marx et al., 2004; Barry et al., 2007; Harris and Thiele, 2011). One interesting question is whether the observed oscillatory shifts are dependent on (lack of) light input or eye closure per se Findings from resting state studies have been contradictory, with reports that α power is modulated by light input but not eye closure itself, and vice versa (Ben-Simon et al., 2013; Jao et al., 2013). Future research should investigate how light input impacts the interaction between eye closure and oscillatory dynamics during active tasks.

### Eye closure versus anticipatory attention

Although eye closure led to a general increase of α power, we found a significant reduction of anticipatory visual α modulation in comparison to the EO condition, with the maxima of this latter phenomenon extending more anterior than the widespread α increase. Somatosensory α lateralization was not affected by eye closure. These observed α modulations are in line with the proposal that α power reflects a functional mechanism of inhibition (Klimesch et al., 2007; Jensen and Mazaheri, 2010; Foxe and Snyder, 2011; Haegens et al., 2011) that regulates cortical excitability to gate information from task-irrelevant regions (here, visual and ipsilateral somatosensory cortices) to task-relevant ones (contralateral somatosensory cortex).

To our knowledge, only two previous studies investigated the interaction between eye-closure-induced and task-related α modulations. Both studies, using auditory paradigms without a spatial component, reported an eye-closure-related increase in α power (Bastarrika-Iriarte and Caballero-Gaudes, 2019; Wöstmann et al., 2020). Wöstmann et al. (2020) found that eye closure enhances the attentional modulation of α power, and Bastarrika-Iriarte and Caballero-Gaudes (2019) found that eye closure enhances the event-related α power increase. Neither study found an effect of eye closure on performance (i.e., accuracy). In their study, Wöstmann et al. (2020) presented to-be-attended and to-be-ignored speech streams binaurally, i.e., attention was equally distributed across auditory cortices. Importantly, they found that eye closure enhances attentional modulation primarily in non-auditory (task-irrelevant) parieto-occipital regions. This mirrors our finding that eye closure only impacts anticipatory visual (task-irrelevant) α modulation. Note that since somatosensory demands are equivalent across eye conditions, and any non-lateralized effects are subtracted out in our lateralization index, it follows that anticipatory somatosensory α remains unaffected by eye closure.

We found that both absolute and anticipatory visual α increase were associated with faster and more accurate responses in both eye conditions. This aligns with our previous findings in the somatosensory (Haegens et al., 2010, 2012) and the auditory domains (ElShafei et al., 2018), demonstrating that in non-visual tasks, visual α increase facilitates behavioral performance. In addition, we found that anticipatory somatosensory lateralization was associated with faster responses, regardless of eye condition. The absence of an effect of somatosensory lateralization on accuracy contradicts our previous findings that lateralization leads to better accuracy (Haegens et al., 2011, 2012). However, a key difference with our current study is the presence of distracting (competing) tactile stimuli in our previous work. If α controls inhibition, it is conceivable that the link between somatosensory lateralization and accuracy is to a degree dependent on the presence of distracting somatosensory stimuli that require suppressing, and we may therefore not have been as sensitive to such effects here.

**Figure 7.**
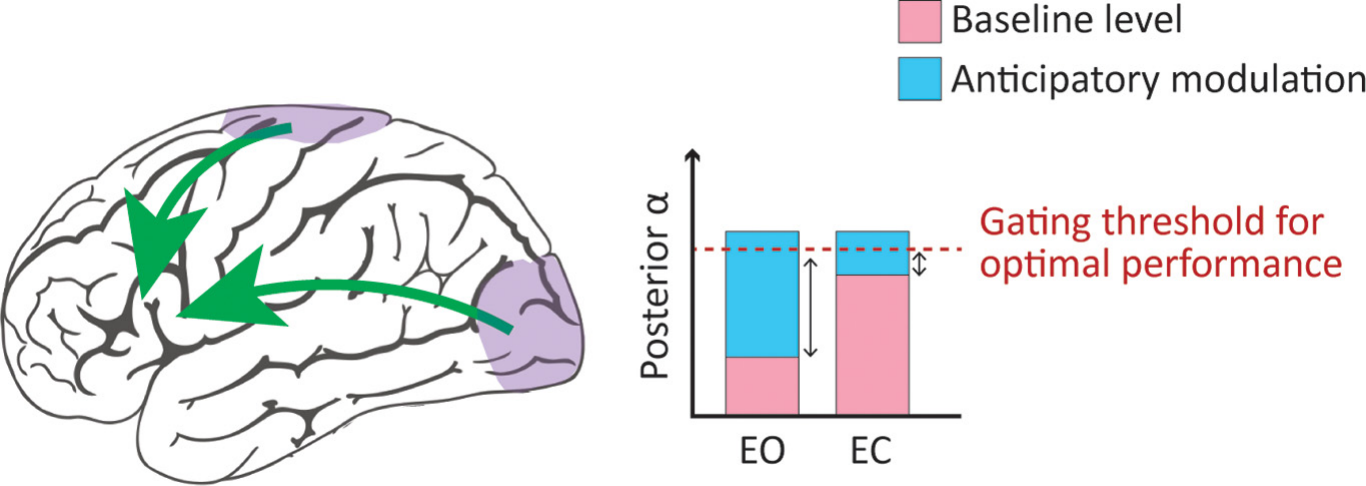
Information gating and eye closure. In the EO baseline interval, information processing is equivalent across task-relevant somatosensory and task-irrelevant visual regions. Thus, in the prestimulus interval anticipatory modulation drives α levels to the optimal gating threshold at which information flow is gated away from visual regions by inhibiting the processing of visual input. In the EC baseline interval information processing is already diminished because of the absence of visual input. However, α level has not yet reached the optimal threshold to entirely gate information flow. Thus, in the prestimulus interval, α level is further heightened to reach the gating threshold and thus inhibiting information processing in visual regions. Please note green arrows indicate general information flow rather than information flow to a certain region.

Critically, all observed α-performance correlations were independent of eye-closure condition, i.e., eye closure did not impact the relationship between α dynamics and behavioral performance. Furthermore, both overall and anticipatory visual α changes showed similar relationships with task performance, suggesting a general (functional inhibitory) role for α, regardless of driving/modulatory factor behind the observed α dynamics. We propose that visual α reflects the inhibition of task-irrelevant visual processing, and that in the presence of visual input (EO condition) an increase in visual α power is required to achieve this, while in the absence of visual input (EC condition), visual α power is already elevated, hence reducing the need for additional anticipatory modulation (Fig. 7). This optimal visual α level might coincide with either a plateau, i.e., a physiological maximum beyond which increases in α levels are not possible, or the peak of an inverted U-shape relationship (between α and performance) beyond which increases in α level could be detrimental.

In conclusion, the present study dissociates for the first time eye-closure-induced α and anticipatory α modulations in the somatosensory domain. We demonstrate that while eye closure boosts overall α power, it dampens anticipatory visual α modulation with no impact on somatosensory lateralization. Finally, we show that eye closure does not alter the impact of α dynamics on behavioral performance. Combined, this suggests there is an optimal visual α level for somatosensory task performance, which can be achieved both through eye closure and top-down anticipatory attention. Our findings provide further support for a general inhibitory or gating role for the α rhythm.

## Synthesis

Reviewing Editor: Satu Palva, Helsingin yliopisto Decisions are customarily a result of the Reviewing Editor and the peer reviewers coming together and discussing their recommendations until a consensus is reached. When revisions are invited, a fact-based synthesis statement explaining their decision and outlining what is needed to prepare a revision will be listed below. The following reviewer(s) agreed to reveal their identity: Andrew Quinn.

The authors conducted an MEG study to contrast behavioural performance and alpha oscillatory power in a spatial tactile discrimination task, which participants (N = 33) performed with open and closed eyes. The results showed that tactile discrimination performance decreased with closed versus open eyes. In the MEG, overall alpha (and beta) oscillatory power increased with closed eyes. At the same time, the anticipatory modulation of alpha power was reduced with closed eyes. Finally, the hemispheric lateralization of alpha power (for trials with attention to the left versus right side) did not differ for open versus closed eyes. Regardless of eye closure, the relation between alpha power (absolute level and anticipatory modulation) to behaviour was the same. The authors incorporate findings into a model wherein baseline and anticipatory alpha modulation explain optimal task performance levels.

The experiment is well-designed and the study is adequately powered. The article is easy to follow and the results are presented in a comprehensive way. The findings are important for our understanding of how neural oscillations shape perception and behaviour. This study is a welcome bridge between two alphas - somatosensory alpha and visual/occipital alpha. It is a well-constructed

piece of work which will be of substantial interest within its field.

Comments

The authors show that eye-closure impairs tactile perception & leads to widespread increases (mainly occcipital) oscillatory activity. Though occipital alpha is higher when a participants eyes are closed, this does not modulate the anticipatory-somatosensory alpha power or its interaction with behavioural performance. The attentional modulation and EC alpha are spatially distinct.

The occipital beta response in Figure 2 seems a little out of place - Could this be a harmonic of the alpha response? This is an important distinction for the theoretical interpretation and would be worth checking by looking at whether that alpha and beta responses have a consistent phase relation. If so - this could reflect a non-sinusoidal alpha response, if not - it could indicate two distinct responses in occiptal cortex.

Most statistical analyses use paired t-tests to explore whether a particular condition A vs condition B difference (across trials within a participant) was consistent across participants - this makes sense but doesn’t explore whether similar effects occur between participants. For example - the analysis shows that absolute visual alpha power is higher in correct trials than incorrect trials, is it then also the case that participants with higher visual alpha power were also correct more often?

The discussion states that more occipital/visual alpha is beneficial as it inhibits visual input processing and that there might be an ’optimal gating threshold’. As there are many known between-subject correlates of alpha power, it would be useful to know if you can show that, or would expect that, your framework extends to this level. eg., do people with low alpha power require larger modulations to reach threshold etc?

Introduction: “alpha decreases contralateral to the attended location (Sauseng et al., 2005) and increases contralateral to the ignored location, presumably to suppress distracting input (Kelly et al., 2009; Worden et al., 2000).” --> There have been a few recent studies which tested the presumed role of lateralized alpha power for distractor suppression more directly (e.g., Wostmann et al. 2019. J Neurosci). The authors might want to refer to this and other related studies here.

Methods: “Finally, for the resulting data, outlier trials of extreme variance were removed” --> It was not entirely clear how outliers were defined.

It is not entirely clear whether the light was switched on or off during the experiment?

Results: “Note that differences in sleepiness scores did not correlate with differences in behavioural performance between eye conditions (RT: r(26) = -0.19, p = 0.32; accuracy: r(26) = 0.22, p = 0.25).”

--> Arguably, the most straight-forward analysis would be to run a mixed-model to predict (single-trial) accuracy or RT from predictors eyes (open vs closed), block order, sleepiness scores, (and maybe trial number). Such a model would help to elucidate whether eye closure, independent of any other effects, modulates performance.

Figure 4B, rightmost panel: “interior” should probably be “inferior"

Figure 7: It remained unclear what the green arrows are supposed to indicate. Especially, why do they point to inferior frontal cortex? While the bar graph in Figure 7 was helpful to integrate the findings into a coherent model, the brain graph (especially including the arrows) was rather unclear.

## Author Response

We are thankful to the reviewers and the editors for reviewing our manuscript and for the positive decision. Below we respond to each of the concerns; we have taken the liberty of numbering them so that they are easier to refer to. In the revised manuscript, modifications are highlighted in yellow. We hope to have adequately addressed all concerns.

1. The occipital beta response in Figure 2 seems a little out of place - Could this be a harmonic of the alpha response? This is an important distinction for the theoretical interpretation and would be worth checking by looking at whether that alpha and beta responses have a consistent phase relation. If so - this could reflect a non-sinusoidal alpha response, if not - it could indicate two distinct responses in occiptal cortex.

This is indeed a very relevant remark for which we have run two control analysis: First, for each participant, we have identified the beta peak frequency within the 15-30 Hz band. We were able to identify distinct beta peaks for all participants (mean = 20.1 Hz {plus minus} 0.26 SEM). Only 11 participants displayed the 1:2 relationship between alpha and beta responses that would indicate a harmonic relationship. In addition, we computed the phase-locking value between alpha and beta signals in order to investigate whether there was a consistent phase relationship within the prestimulus window. Time points were then shuffled in order to create surrogate phase-locking values. As shown in Response Figure 1, we found no differences between the real and surrogate phase-locking values (p = 0.07) using nonparametric cluster-based permutation tests. Taken together, we argue against a harmonic relationship between alpha and beta responses.

Response Figure 1. Real (in blue) and surrogate (in red) phase locking values between alpha and beta responses.

2. Most statistical analyses use paired t-tests to explore whether a particular condition A vs condition B difference (across trials within a participant) was consistent across participants - this makes sense but doesn’t explore whether similar effects occur between participants. For example - the analysis shows that absolute visual alpha power is higher in correct trials than incorrect trials, is it then also the case that participants with higher visual alpha power were also correct more often?

It is indeed important to explore the between-participants effects. We grouped participants according to (1) absolute visual alpha power, (2) anticipatory visual alpha modulation, and (3) somatosensory lateralization index, and then compared accuracy (percentage of correct responses) and median reaction time (RT) between groups using paired t-tests. As shown in Response Figure 2, there were no significant differences between groups.

Response Figure 2. Accuracy (upper row) and median reaction times (lower row) for participants with low and high absolute visual alpha power, anticipatory visual alpha modulation, and somatosensory alpha lateralization.

3. The discussion states that more occipital/visual alpha is beneficial as it inhibits visual input processing and that there might be an ’optimal gating threshold’. As there are many known between-subject correlates of alpha power, it would be useful to know if you can show that, or would expect that, your framework extends to this level. eg., do people with low alpha power require larger modulations to reach threshold etc?

As we suggested in the discussion, we would indeed expect the amplitude of visual modulation to vary with the individual optimal gating threshold, which should also vary across individuals. However, the relationship between the individual gating threshold and the alpha level could be either direct or inverse, i.e., individual low alpha level could correspond to a lower gating threshold (and subsequently lower modulation), or low alpha level could correspond to a higher gating threshold (and subsequently higher modulation).

Since we did not collect resting state data, it is hard to dissociate an individual resting alpha level from ongoing alpha levels as the latter is part of the visual modulation computation. Nevertheless, for each eye condition, we sorted participants according to their alpha power during the baseline period (i.e., pre-cue) and then compared the amplitude of their visual alpha modulation using a repeated-measures ANOVA. As shown in Response Figure 3, we found that visual alpha modulation was higher in the eyes-open condition in comparison to the eyes-closed (p < 0.001). In addition, we demonstrate that visual alpha modulations were higher for participants who displayed higher baseline alpha levels (p = 0.021). Thus, it seems that that visual modulation and baseline alpha levels are positively related.

Response Figure 3. Anticipatory visual alpha modulation (baseline corrected) for participants with low and high baseline visual alpha power. * p < 0.05, *** < 0.001.

4. Introduction: “alpha decreases contralateral to the attended location (Sauseng et al., 2005) and increases contralateral to the ignored location, presumably to suppress distracting input (Kelly et al., 2009; Worden et al., 2000).” --> There have been a few recent studies which tested the presumed role of lateralized alpha power for distractor suppression more directly (e.g., Wostmann et al. 2019. J Neurosci). The authors might want to refer to this and other related studies here.

Thank you for pointing out the missing reference which has now been added to the manuscript.

5. Methods: “Finally, for the resulting data, outlier trials of extreme variance were removed” --> It was not entirely clear how outliers were defined.

We have modified the text to clarify outlier exclusion method. Trials were visually inspected and rejected if their amplitude was higher than two standard deviations from the average of all trials.

6. It is not entirely clear whether the light was switched on or off during the experiment?

We have modified the text to clarify that the magnetic shield room was dimly lit throughout the entire experiment.

7. Results: “Note that differences in sleepiness scores did not correlate with differences in behavioural performance between eye conditions (RT: r(26) = -0.19, p = 0.32; accuracy: r(26) = 0.22, p = 0.25).” Arguably, the most straight-forward analysis would be to run a mixed-model to predict (single-trial) accuracy or RT from predictors eyes (open vs closed), block order, sleepiness scores, (and maybe trial number). Such a model would help to elucidate whether eye closure, independent of any other effects, modulates performance.

According to the reviewer’s suggestion we ran a mixed model to predict behavioural measures (accuracy and reaction time) from eyes, block order and sleepiness. The input for the model was these respective measures averaged across blocks (as participants rated their sleepiness after each block). Sleepiness scores could not predict neither participants reaction time (p = 0.99) nor accuracy (p = 0.07).

8. Figure 4B, rightmost panel: “interior” should probably be “inferior"

Text in panel has been changed accordingly.

9. Figure 7: It remained unclear what the green arrows are supposed to indicate. Especially, why do they point to inferior frontal cortex? While the bar graph in Figure 7 was helpful to integrate the findings into a coherent model, the brain graph (especially including the arrows) was rather unclear.

We have modified the text in the legend for Figure 7 to mention that green arrows indicate general information flow rather than information flow to a certain region.
